# Peer review processes and related issues in scholarly journals

**DOI:** 10.1186/s40199-015-0099-4

**Published:** 2015-02-24

**Authors:** Soodabeh Saeidnia, Mohammad Abdollahi

**Affiliations:** Medicinal Plants Research Center, Tehran University of Medical Sciences, Tehran, 1417614411 Iran; Faculty of Pharmacy and Pharmaceutical Sciences Research Center, Tehran University of Medical Sciences, Tehran, 1417614411 Iran

## Introduction

The determination as to whether a manuscript should be refused or accepted is an important job of the editors that is accomplished through a “peer review” procedure. This procedure dates back to the 17th century, although it was not given to all submissions by medical journals until the mid–20th C. Referable to the elaboration of science and specialization, and besides the impulse to publish among academics, the number of articles has increased, so journals need to select the highest quality manuscripts from the large number of submissions for publication [1]. For this reason, the peer review process has evolved and grown up besides other skills like typing. Although, nowadays some famous and high ranked journals hire expert editors and staff who supervise the review process [2], they don’t accept articles only after in-house review. In some cases, editors reject articles after in-house review. However, most journals do not have a large staff and therefore send almost everything to external reviewers. In that location is an imagination that the invitation for review from high-ranked journals has usually been accepted by the reviewers, while they may be loath to review for smaller or ordinary journals. Nonetheless, even big journals complain that reviewers often refuse to perform a recap.

The general routine in most journals is that the editor in chief or the editorial team appraise all submissions, and then separate them into suitable or unsuitable manuscripts regarding the scope and strategy defined previously for the journal. Thus, in this step, many submissions might be declined or rejected. After that, suitable submissions are sent out to external reviewers. Finding interested and professional scientists or investigators to accept the review invitation is not easily carried out and sometimes is a difficult process. Because of this problem, the range of reviewers is reduced and the journals may not have enough choices to replace reviewers who perform poorly or slower.

## Importance of an acceptable review

**Figure 1:**
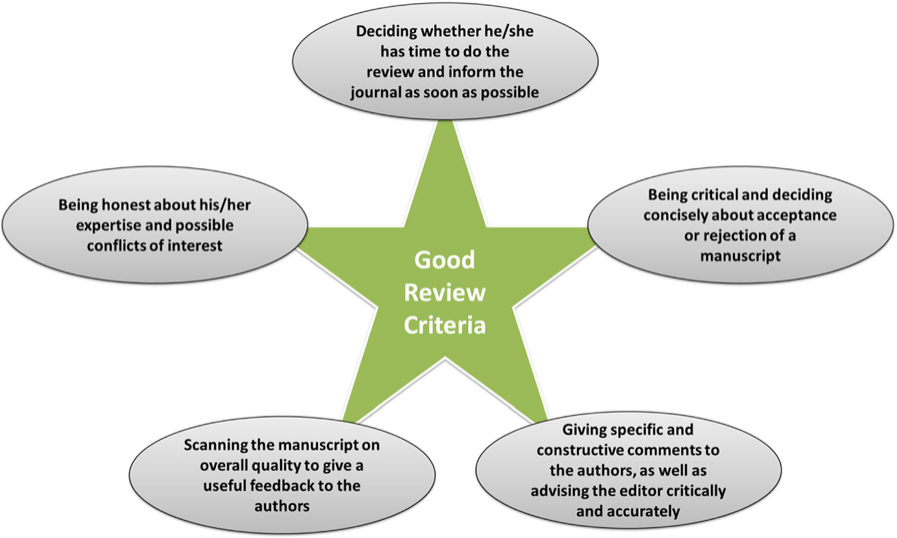
**Some important criteria and parameters, which good reviewers should follow** **[** 3 **]** **.**

**Figure 2:**
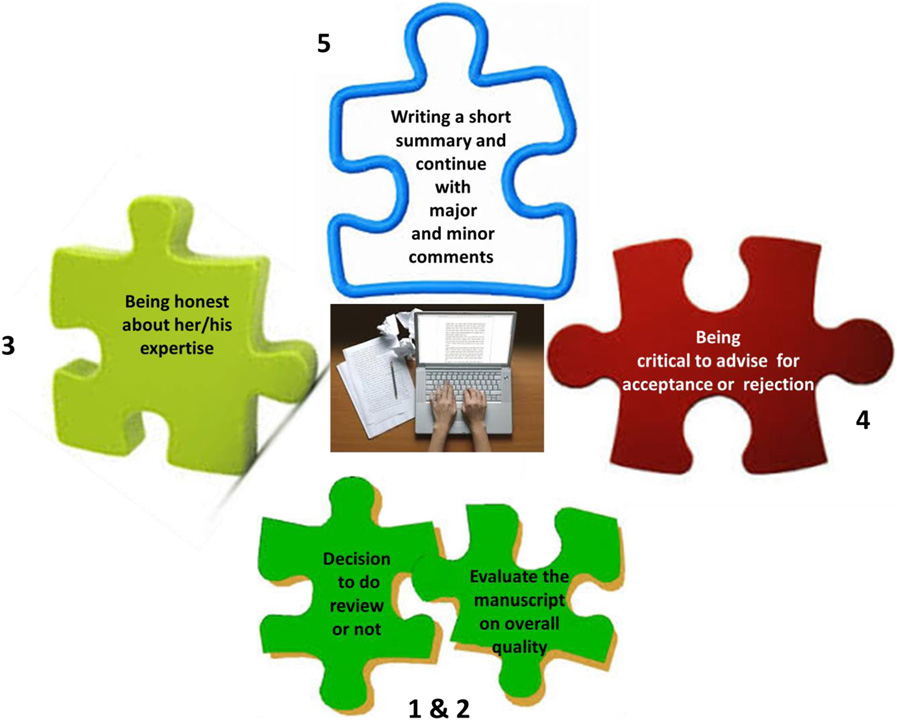
**General guidance for reviewers to start a review** **[** 3 **].**

When a reviewer (external or internal) exhibits a good or acceptable review, his or her advice can influence the editor’s decision on selection of the best manuscripts for publishing. In addition, a critical review process is able to make a submission better and add to its transparency and accuracy for the readers. Today, the query is: “Who is a good reviewer?” or “What is a good peer review process?” According to a literature review on this topic and also personal experience, there are a bit of criteria by which a good reviewer is identified. Some of these are summarized in Figure 1 [3]. Likewise, a schematic guidance that reviewers can generally use is indicated in Figure 2. Ideally, peer review should be a highly objective, honest, and consistent procedure, in which writers and editors can trust. Thus, to reduce the adverse effects of peer review, journals and editors have adopted systems such as single or two blinded review models, clarifying the peer review process on the web, as well as training reviewers [4].

Regrettably, there have been only a few surveys in which the peer critique process in diverse journals has been assessed or statistically analyzed. A reason might be ascribable to the complexity of this challenging operation. The limited available documents show that a good peer review process has to discover a path toward improving the reporting quality of published papers [3,5]. People who create poor quality reviews may be removed from the database, especially if editors rate reviewers’ performance [3].

## Common drawbacks of peer review process

Peer review has its own defects such as being slow and unreliable, not necessarily objective and consistent, sometimes displaying bias against certain authors, or even misuse such as stealing the ideas or applying a critical review to halt or at least slow down publications by rivals [4]. Likewise, the originality of a study is assumed to be examined by the reviewers, which is sometimes given out to meet. Some reviewers have even claimed that they were not mindful of that responsibility supposing the investigation was a task of the editorial panel.

In the single review model, disclosing the author’ name may sometimes, intentionally or unintentionally affect the decision of reviewers in a positive or negative aspect. The reviewers should be trained to report objectively with more attention to context. This is specially important in bio-medical specialty and pharmaceutical journals, as the results of bias might be harmful.

## The negative results are usually not welcome

The reviewers may be biased when they see reports of negative information. Not only authors may avoid publishing negative results, but also editors and reviewers might think that such resolutions will be considered too dull to read by the journal readers. This bias should be taken into account by scientists in all the roles of author or editor or reviewer.

## A time consuming process

Publishing in scholarly journals usually takes time to complete internal and external assessment of the articles. But sometimes these tasks are done with a delay originated from the journal editorial office or reviewers or the editors. Most of the time, delays originate from the time required to get a suitable reviewer who agrees to review and then by the reviewers who fail to complete the review on time. The reviewers should be selected from the professions who are reliable and well-known in the field of study and adhere to ethical rules. But there is no such guarantee to find the best reviewers who could meet the required journal desires. Therefore, this part of the task is always complicated [6].

## Self-citation request by reviewers

It has been observed that sometimes peer reviewers request authors to cite their publications, either appropriately or via compulsive self-citation to highlight the reviewers’ work. In order to evaluate this circumstance, peer reviews for manuscripts submitted in 2012 to the Journal of Psychosomatic Research were evaluated. Totally, there were 616 peer reviews (526 reviewers; 276 manuscripts), of which 444 recommended for revision or acceptance and 172 rejected. Surprisingly, the results showed that there were 122 peer reviewer self-citations (29%) and 306 citations to others’ work (71%) of 428 total citations. Furthermore, it was observed that self-citations existed generally in the review articles which were recommended for revision or acceptance (105 of 316 citations; 33%) compared to rejection (17/112; 15%; p < 0.001). The authors concluded that the percentage of self-citations with no rationale (26 of 122; 21%) was higher than for citations to others’ work (15 of 306; 5%; p < 0.001) [7].

## A must to do for young reviewers

Offering a thorough review is not easy, particularly for new reviewers, who simply receive an invitation letter from a journal with short or no counselling or preparation. The reports should be clear, concise, and exact. The report should aid the editor decide whether to admit the manuscript (the main purpose) and the comments to the authors should help them improve the manuscript even if they are rejected and sent to a different journal. Usually, comments for authors are briefly written especially when the reviewer feels the paper has valuable points, but needs major revision to deserve publication. Regrettably, there are reviewers who attempt to conceal behind the anonymity and write a critical report (snide, sarcastic, argumentative even obscene) that should be censored before being transmitted to the authors. Surely, such gratuitous rudeness and personal criticism have never been more appropriate. Reviewers are urged to write their report using an acceptable manner and timbre that they would wish to meet if they were in the position of author [8].

Efficiency of training the reviewers had been already assessed by a randomized trial, in which three groups of reviewers were included: 1- who got nothing; 2- who had a daily face-to-face training plus a CD-ROM; 3- got only the CD-ROM. The results showed some improvements associated with those who had training, although the consequences of various groups did not show a significant difference [9]. It indicated that the primary trouble with the study was that many of the reviewers were already an expert, so it is possible that younger reviewers would respond more to training [9].

## Conclusion

In the big world of science, researchers, academics and students are all under pressure to publish more and in order to meet the ever-increasing publication activities, thousands of new publishers have sprung up globally, and the number of online and subscription journals has increased exponentially [10]. But the determination of the suitability or unsuitability of a manuscript needs a “peer review” procedure. Although the peer-review system is a valuable instrument to pick out the best projects and ideas and publish them, it faces some criticisms and concerns, including: the process may wrongly reject scientifically valid papers, or on the other hand, it may wrongly accept scientifically flawed papers, and may be biased against non-majors or negative point of views. Basic principles, to which peer reviewers should adhere, described by the COPE and summarized in Table 1. Decreasing the faults and avoiding such errors is not possible, whereas it is possible to improve reviewers by pursuing efficient training to teach principles and ethical guidance [11].

**Table 1:** **Basic principles to which peer reviewers should adhere, described by the COPE, and the related issues**

**COPE Ethical Guidelines for Reviewers** ^**1**^	**Issues**	**Comments**
Only agree to review manuscripts for which they have the subject expertise required to carry out a proper assessment and which they can assess in a timely manner	Lack of expert reviewers to accept to do a review.	Training the young reviewers in academic levels in various fields of expertise.
Respect the confidentiality of peer review and not reveal any details of a manuscript or its review, during or after the peer-review process, beyond those that are released by the journal	The relationship between publications/journal editors and institutions.	Blind review may reduce it, especially when a journal is related to an institution.
Not use information obtained during the peer-review process for their own or any other person’s or organization’s advantage, or to disadvantage or discredit others	Getting information for own might be rare, but occurred.	This can be reduced, if the journal appears the name of reviewers too.
Declare all potentially conflicting interests, seeking advice from the journal if they are unsure whether something constitutes a relevant interest	Some reviewers might declare nothing while there is.	Reviewers should be trained to know what is assumed as conflict; Keeping full CV of reviewers for emergency cases; Appearing the name of the reviewers.
Not allow their reviews to be influenced by the origins of a manuscript, by the nationality, religious or political beliefs, gender or other characteristics of the authors, or by commercial considerations	Young authors, women, colleagues of not famous institutions and developing countries are usually suffering from such reviewing.	Blind review reduces the cases; Selecting reviewers from similar locations or countries may be effective.
Be objective and constructive in their reviews, refraining from being hostile or inflammatory and from making libelous or derogatory personal comments	This may happen when the reviewer has not enough experiences, or when he/she wants to use the information for own (or institution), or there is a conflict of interest too.	Blind review reduces the cases; Declaration of conflicts is effective to avoid; Training reviewers that what is expected from a peer review process.
Acknowledge that peer review is largely a reciprocal endeavor and undertake to carry out their fair share of reviewing and in a timely manner	This may happen when the reviewer is busy without appropriate time to do a review; Some academics are urged to do review due to the academic tasks or only for promotion.	Explaining to reviewer about the importance of being on time; Considering some opportunities for on time reviewers like access to scientific databases.
Provide journals with personal and professional information that is accurate and a true representation of their expertise	-	Training reviewers to know the code of ethics in the peer review process.
Recognize that impersonation of another individual during the review process is considered serious misconduct	Rare cases.	Blind review reduces the cases; Declaration of conflicts is effective to avoid; Training reviewers to know the code of ethics in the peer review process.

^1^Irene Hames on behalf of COPE Council, COPE Ethical Guidelines for Peer Reviewers, March 2013, v.1, publicationethics.org [11].
